# Significance of PPARA as a Treatment Target for Chronic Lymphocytic Leukemia

**DOI:** 10.1155/2023/8456833

**Published:** 2023-06-26

**Authors:** Xixi Xiang, Fu Li, Sha Zhou, Yunjing Zeng, Xiaojuan Deng, Hongyang Zhang, Jiali Li, Hongyun Liu, Jun Rao, Lei Gao, Cheng Zhang, Qin Wen, Li Gao, Xi Zhang

**Affiliations:** State Key Laboratory of Trauma, Burns and Combined Injury, Medical Center of Hematology, Xinqiao Hospital of Army Medical University, Key Subject of Chongqing, Chongqing 400037, China

## Abstract

Peroxisome proliferator-activated receptor alpha (PPARA) has been suggested as a therapeutic target for chronic lymphocytic leukemia (CLL). However, the underlying molecular mechanism remains largely unclear. In this study, we analyzed DNA next-generation sequencing (NGS) data and clinical information from 86 CLL patients to identify gene markers related to treatment-free survival (TFS) length. We then constructed a genetic network that includes CLL promoters, treatment targets, and TFS-related marker genes. To assess the significance of PPARA within the network, we utilized degree centrality (DC) and pathway enrichment score (EScore). Clinical and NGS data revealed 10 TFS length-related gene markers, including RPS15, FOXO1, FBXW7, KMT2A, NOTCH1, GNA12, EGR2, GNA13, KDM6A, and ATM. Through literature data mining, 83 genes were identified as CLL upstream promoters and treatment targets. Among them, PPARA exhibited a stronger connection to CLL and TFS-related gene markers, as evidenced by its ranking at No. 13 based on DC, compared to most of the other promoters (>84%). Additionally, PPARA co-functions with 70 out of 92 in-network genes in various functional pathways/gene groups related to CLL pathology, such as regulation of cell adhesion, inflammation, reactive oxygen species, and cell differentiation. Based on our findings, PPARA is considered one of the critical genes within a large genetic network that influences the prognosis and TFS of CLL through multiple pathogenic pathways.

## 1. Introduction

Chronic lymphocytic leukemia (CLL) is a tumor originating from mature B lymphocytes [1]. It is the most common adult leukemia in Western countries [2]. In 2021, about 21,250 people in the United States were diagnosed with CLL, and about 4,320 died from this disease [3]. The clinical course of patients with CLL is highly heterogeneous, making it difficult to predict the likelihood that a patient will require treatment at the time of diagnosis. About 70%–80% of CLL patients are asymptomatic at the time of diagnosis, and around 30% will never require treatment for CLL [4]. Clonal diversity and complexity have consistently been associated with poor CLL prognosis [5, 6].

Nuclear receptor peroxisome proliferator-activated receptor alpha (PPARA) is a critical regulator of energy metabolism and mitochondrial and peroxisomal function [7]. Encoded by the PPARA gene, PPARA regulates the expression of genes involved in glucose and lipid metabolism and inflammatory processes [8] by binding to PPAR response elements in the promoter region of the genes [9]. PPARA has been shown to mediate glucocorticoid resistance and promote CLL [10] and has therefore been suggested as a therapeutic target for CLL [11]. A PPARA antagonist, NXT629, demonstrated the capability of reducing tumor burden in a mouse model of CLL [12], suggesting that PPARA may have a tumor-suppressive role in CLL. However, the precise mechanism by which PPARA regulates CLL is yet to be determined.

Besides PPARA, there are multiple genes that have been identified to play roles in the survival of CLL B cells and were suggested as therapeutic targets in CLL, including NOTCH [13, 14], ZAP70 [15], CXCR4 [16], CD40 [17], CD44, and CD49d [18]. These genes could influence the prognosis of CLL patients and their treatment-free survival (TFS) length. It is worth noting that the proposed therapeutic targets for CLL, including PPARA, have been suggested by different studies conducted at various time intervals. Identifying and analyzing the relationship among these genes may help discover their significance and better understand their therapeutic roles in treating CLL [19].

In this study, we collected TFS-related gene markers by using DNA next-generation sequencing (NGS) data and clinical data collected from 86 CLL patients. We constructed the genetic network composed of the literature-identified CLL promoters and treatment targets, as well as CLL TFS-related marker genes. Degree centrality (DC) and pathway enrichment score (EScore) were used to evaluate the significance of each gene within the genetic network, including PPARA.

## 2. Materials and Methods

### 2.1. Patient Samples and Laboratory Testing

A total of 86 newly diagnosed CLL patients from Chongqing and other regions in western China from January 2019 to June 2021 in the Hematology Medical Center of Chongqing Xinqiao Hospital were collected. According to the iwCLL 2018 diagnostic criteria [20], these patients were diagnosed as CLL. The clinical and laboratory characteristics of all patients were collected, including gender, age, imaging examination, blood routine test, biochemical test, flow cytometry, and chromosome karyotype. Fluorescence in situ hybridization (FISH) image was used to detect del(17p), del(11q), and del(13q). Sanger sequencing of Polymerase Chain Reaction (PCR) amplification products was used to determine the immunoglobulin heavy chain variable region (IGHV) mutation status. Peripheral blood (*n* = 39) and bone marrow aspirate (*n* = 47) samples from 86 untreated CLL patients were collected for DNA–NGS data generation. According to the guidelines, patients without indications for treatment were followed up regularly, and appropriate treatment was given after reaching time-to-first treatment (TTFT). The TTFT for CLL patients is the time between diagnosis and the initiation of the first treatment and can vary depending on several factors. Close monitoring and appropriate treatment can help improve outcomes for patients with CLL. Patients are treated in a combination of hospital visits and follow-ups on the Internet management platform for CLL patients in the Hematology Medical Center of Chongqing Xinqiao Hospital. This study was approved by the Ethics Committee of the Second Affiliated Hospital of Army Medical University (2020-Research No. 128-01).

### 2.2. NGS Data Collection

In this study, the CLL-related gene mutation detection kit (CLL 72) (Wuhan Steady Medical Laboratory Co., Ltd.; http://www.stdlbio.com/) was used to target 72 CLL-related genes based on NGS technology. The 72 genes cover chronic lymphoproliferative disease genes associated with Chinese onset recommended by authoritative guidelines/expert consensus at home and abroad, large-scale literature reports, and database accumulation, and are used to assist in routine screening of clinical CLL.

First, DNA extracted from blood and bone marrow samples was used as material to prepare a pre-library by fragmentation, adapter addition, PCR amplification, and other steps; then, biotin-labeled oligonucleotide probes were used to hybridize with the pre-library, and then Streptavidin magnetic beads are used to bind the probe to capture the target region; finally, the final capture library is obtained by PCR enrichment. The amplified library was sequenced using a gene sequencer (model: MiniSeq, MiSeq, NextSeq, NovaSeq, etc., produced by Illumina Corporation) for high-throughput sequencing.

Trimmomatic (v0.39) software was used to filter the raw data for quality control, and the filtered data were compared to the human reference genome (GRCh37) through the BWA-mem (v0.7.17) algorithm. Then, GATK (4.0.12.0) software was used to detect mutation sites, and Annovar (v20210122) software was used to annotate the mutations in combination with databases such as NCBI, COSMIC, Clinvar, ExAC, dbSNP (v138), and 1000 Genomes.

### 2.3. Statistical Method

Data analysis was performed using IBM SPSS Statistics 26. The measurement data conforming to the parametric distribution were expressed as the mean ± standard deviation; otherwise, the median (interquartile range) was expressed, and the enumeration data were expressed as the number of cases and percentages. TTFT was defined in months, from the date of initial diagnosis to the date of initial treatment. For all surviving patients, the time of statistical analysis by the researchers (March 2022) was taken as the last follow-up time. The COX risk regression model was used to evaluate the correlation analysis between patient characteristics and TTFT. First, the data were analyzed by univariate analysis, and the factors with univariate analysis *P* < 0.1 were included in the multivariate model for stepwise regression analysis. We reported and conducted further analysis on the factors that demonstrated significance (*P* < 0.05) regarding their association with gene mutations. R-4.1.2 was used for survival analysis and Kaplan–Meier curve drawing. Use the chi-square test function in R-4.1.2 to detect differential genes, and *P* < 0.05 criteria was used to determine the statistically significant genes related to TFS.

### 2.4. Construct the Genetic Therapeutic Network for CLL

Assisted by Pathway Studio (http://www.pathwaystudio.com), we conducted a literature data mining to uncover reported CLL therapeutic target genes and positive upstream regulators. The genes that were identified in previous studies as potential therapeutic or treatment targets for CLL are referred to as the “therapeutic targets.” On the other hand, genes that have been shown to positively regulate CLL based on previous research are known as the “positive upstream regulators.” To gather information on these genes and their relationship with CLL, a literature data mining approach was employed using a comprehensive biology database (http://www.pathwaystudio.com) that covers the entire PubMed abstracts and full-text journals from Elsevier and third-party publishers. This database is one of the largest in the world. For each gene–CLL relationship, there are one or more supporting references that were manually reviewed for quality control.

Then we constructed the network connection to these genes, CLL, PPARA, and the TFS-significant genes identified from the NGS data and clinical data analysis.

### 2.5. Weighting PPARA in the Therapeutic Network of CLL

In graph theory, a vertex's centrality reflects the vertex's significance in the network. The simplest vertex centrality is DC, defined as the number of edges incident upon a vertex. For a graph *G*: = (*V*, *E*) with *n* vertices, the DC *C*_D_(*v*) for vertex *v* is defined in Equation (1).

(1) $$\begin{array}{c} C_{\text{D}}\left(v\right)=\frac{\text{Degree}\left(v\right)}{n-1}. \end{array}$$

Besides DC, we also employed the EScore [21], which reflects a gene's importance in the significant pathways involved. The Escore for the “*v*th” gene is calculated as Equation (2).

(2) $$\begin{array}{c} {\text{EScore}}_{v}=\frac{\underset{i=1:m}{\text{sum}}\left(-\log_{10}\left(p_{i}\right)\right)}{\underset{i=1:n}{\max}\left(-\log_{10}\left(p_{i}\right)\right)}, \end{array}$$

where *p*_*i*_ is the enrichment *p*-value of the “*i*th” pathway enriched, “*n*” represents the total number of pathways enriched, and “*m*” represents the number of pathways including the “*v*th” gene.

## 3. Results

### 3.1. Clinical Data of the 86 CLL Patients

The median age of the 86 patients was 57.34 ± 10.81 years old (range 34–81 years old), 61 males (70.9%), and 25 females. CLL-international prognostic index (CLL-IPI) score was high risk (including very high risk) in 12 cases (13.9%). There were 61 cases (70.9%) of IGHV mutations. NGS detected TP53 mutations in five cases (5.8%). FISH detected del(13q) in 12 cases (13.9%), del(11q) in 13 cases (15.1%), del(17p) in 5 cases (5.8%), and 12 amplifications in 2 cases (2.3%). The characteristics of the patients are shown in Table 1. Del(13q) refers to a chromosomal abnormality in which a portion of the long arm of chromosome 13 is missing. This deletion is a common finding in CLL and is the most frequent chromosomal abnormality in this type of leukemia. Del(11q) refers to a chromosomal abnormality in which a portion of the long arm of chromosome 11 is missing. This deletion is commonly found in patients with CLL and is one of the most significant genetic abnormalities associated with poor prognosis in this disease. Del(17p) refers to a chromosomal abnormality in which a portion of the short arm of chromosome 17 is missing. This deletion is also known as 17p deletion or TP53 deletion because it affects the TP53 gene located on this chromosome.

The median follow-up time of all patients was 22.00 months (95% CI 20.48–23.51), 31 cases (36.05%) achieved TTFT (TTFT-24) within 24 months, and the median TTFT (mTTFT) was 5.00 months (95% CI 2.31–7.69). A total of seven patients (8.14%) died, of which two patients who did not reach TTFT died of other causes (cardiovascular events and pulmonary infection) other than disease progression. Richter's transformation (diffuse large B-cell lymphoma) was present in 4 of 31 TTFT-24 patients.

### 3.2. TFS-Related Clinical Parameters and Genes

**Figure 1:**
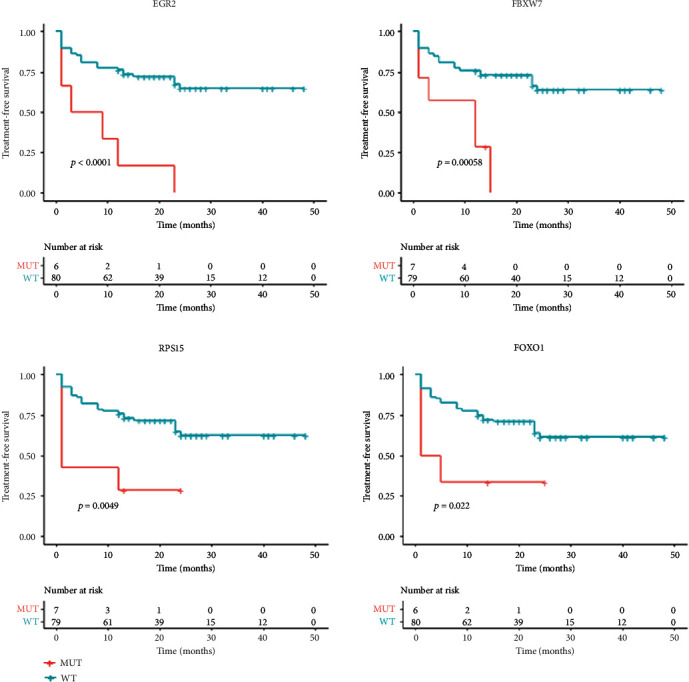
EGR2, FBXW7, RPS15, and FOXO1 mutations were shown to be associated with shorter treatment-free survival (TFS) in 86 patients.

We presented the spectrum of gene mutations of 86 patients in Supplementary Figure 1. DNA-seq data analysis showed that the mutations of four genes presented significant associations with shorter TFS (Figure 1), including EGR2, FBXW7, RPS15, and FOXO1.

The results of multivariate analysis showed that four factors are associated with shorter TFS and can be used as independent prognosis decision factors, including CLL-IPI score (*P* = 0.025, 95% CI 1.14, 7.02), del(11q) (*P* = 0.002, 95% CI 1.62, 8.81), splenomegaly (*P* = 0.009, 95% CI 3.24, 7.08), and newly diagnosed platelets (<100 × 10^9^/L) (*P* = 0.003, 95% CI 1.63, 11.17). In Supplementary Table 1, we presented the analysis results for all factors. However, due to limited space, we only emphasized the discussion on the four significant factors.

**Figure 2:**
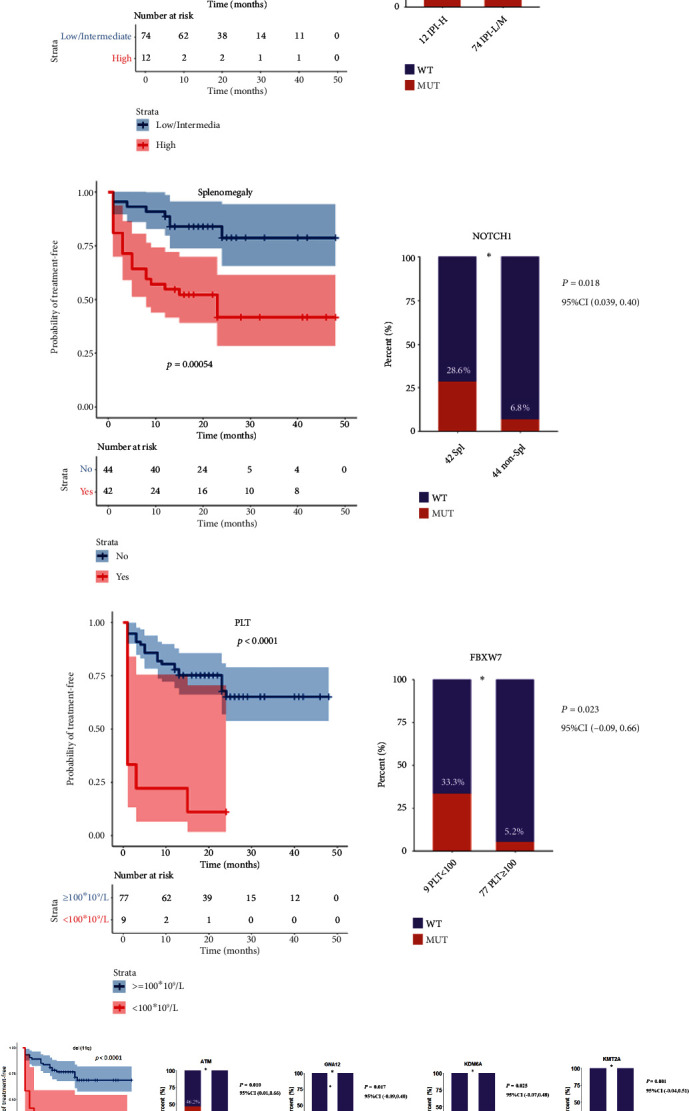
The four clinical factors associated with shorter TFS and their associated genes. (a) CLL-IPI related gene; (b) Splenomegaly related gene; (c) platelets related gene; (d) del(11q) related genes.

The chi-square test showed that NOTCH1 mutation (*P* = 0.048, 95% CI −0.06, 0.62) was linked to the CLL-IPI score. Associated with del(11q) were ATM mutation (*P* = 0.010, 95% CI 0.01, 0.66), GNA12 mutation (*P* = 0.016, 95% CI −0.09,0.39), KDM6A mutation (*P* = 0.025, 95% CI −0.07, 0.48), and KMT2A mutation (*P* = 0.001, 95% CI −0.04, 0.51). The differential gene mutation associated with newly diagnosed patients with platelet <100 × 10^9^/L was FBXW7 mutation (*P* = 0.023, 95% CI −0.09, 0.66). NOTCH1 mutations were associated with splenomegaly (*P* = 0.018, 95% CI 0.039, 0.40). Two gene mutations, EGR2 (*P* = 0.014, 95% CI −0.17, 1.00) and GNA13 (*P* = 0.030, 95% CI −0.31, 0.81), were associated with Richter's transformation. We presented the clinical parameters associated with shorter TFS and their associated genes in Figure 2.

### 3.3. CLL-Regulation Network

**Figure 3:**
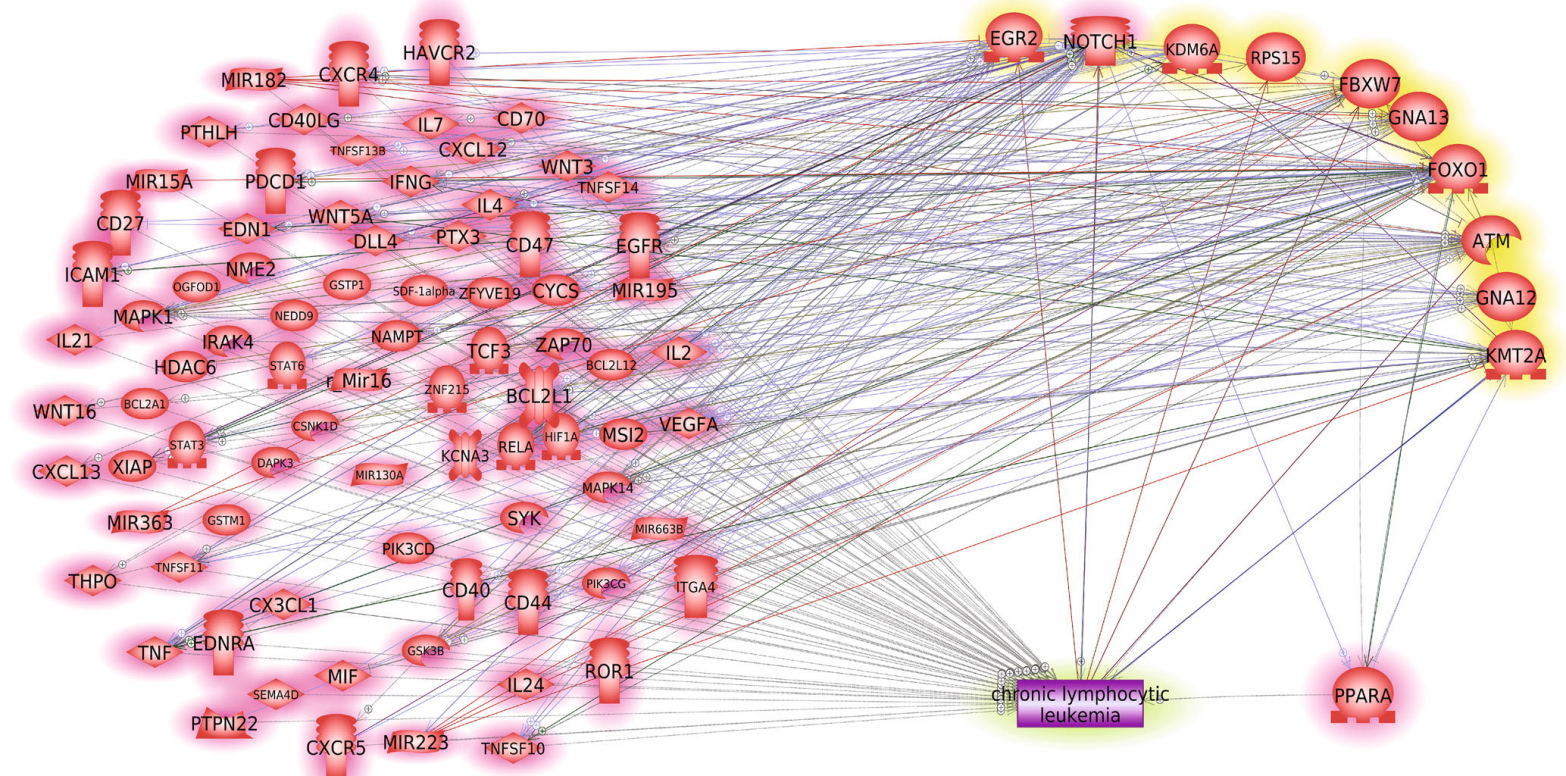
CLL-regulation network composed of 83 CLL upstream promoters (highlighted in red) and 10 CLL TFS-related genes (highlighted in yellow). NOTCH1 was in both groups and highlighted by mixed colors (red and yellow).

Literature data mining identified 83 genes as CLL upstream promoters and treatment targets, including PPARA. Their connections with CLL and the 10 CLL TFS-related genes were employed to construct the CLL-regulation network, as shown in Figure 3. In Supplementary Table 2, we outline each relationship in detail, including the relation type, relation direction, source node, and target node. The 10 genes associated with CLL TFS were marked in yellow, while the 83 CLL upstream promoters, including PPARA, were marked in red. The network was established in such a way that links the 83 CLL upstream promoters to both the 10 CLL TFS-related genes and CLL itself. All the relationships in the network were supported by one or more references. Utilizing this constructed network, we evaluated and compared the centrality scores of each of the 83 CLL promoters to determine their association with CLL and its TFS length.

### 3.4. DC and EScore of PPARA

**Figure 4:**
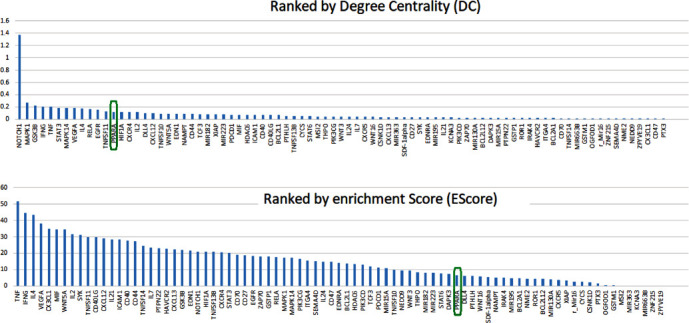
PPAR ranking. (a) Using degree centrality (DC); (b) using enrichment score (EScore).

Ranked by DC, PPARA is the No. 13 in 83 CLL promoters, as shown in Figure 4(a). The results suggest that PPARA presents a tighter connection to CLL and TFS-related gene markers than most (>84%) of the previously reported CLL promoters, indicating the significance of PPARA as a treatment target for CLL.

**Figure 5:**
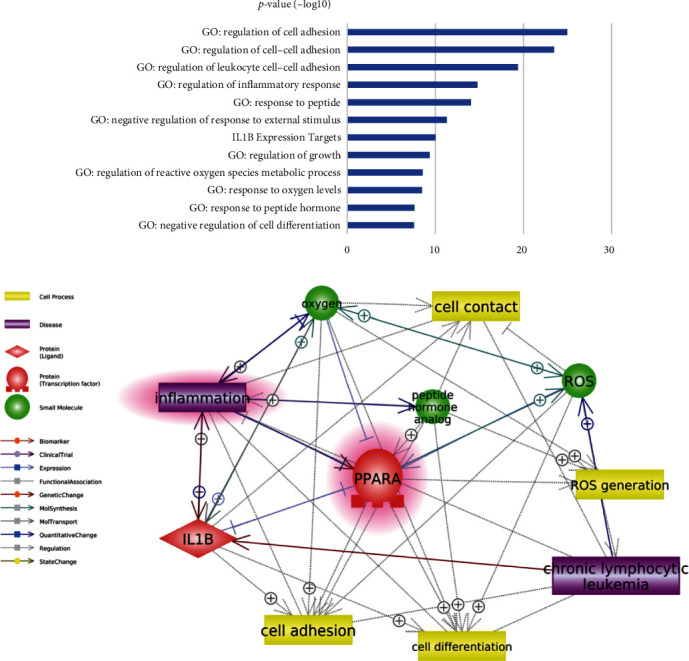
Functional connection between PPARA and chronic lymphocytic leukemia (CLL). (a) The significantly enriched pathways/GO terms involving PPARA; (b) corresponding literature-based pathways connecting PPARA and CLL.

Ranked by EScore, PPARA is indexed as No. 57 out of 83 CLL promoters, as shown in Figure 4(b). The relatively low EScore indicated that the role of PPARA influencing CLL TFS might be mainly focusing on a limited number of functional areas, such as regulation of leukocyte cell–cell adhesion and inflammatory response, as shown in Figure 4. To note, a high EScore signifies that the gene participates in a broader range of pathways, whereas a lower EScore suggests that the gene is more concentrated on specific functional areas. Its importance is determined by not only how many pathways are involved but also how crucial these biological pathways are to CLL. Our investigation revealed that the majority of the pathways enriched by PPARA are crucial in the pathological development of CLL, such as cell adhesion, cell differentiation, and inflammation (as illustrated in Figure 5(b)).  Figure 5 illustrates the role of PPARA in terms of the pathways it is involved in. Specifically, Figure 5(a) depicts the pathways that involve PPARA, while Figure 5(b) shows how these pathways (functional groups) are related to PPARA and CLL, providing a comprehensive overview of the role of PPARA in CLL. It is also worth mentioning that PPAR was functionally linked with most of these 83 genes (70/83 = 84.34%) to play roles in these pathways associated with the pathology of CLL.

## 4. Discussion

Numerous investigations utilizing animal models and human cell lines have explored the link and function of PPARA in CLL. The findings indicate that PPARA promotes the development of CLL, and thus, targeting PPARA gene regulation may serve as a potential therapeutic strategy for managing CLL [10–12, 22]. PPARA was found to be expressed by circulating CLL cells and highly associated with advanced-stage of CLL [11]. In 2015, Messmer et al. developed a PPARA antagonist, NXT629, which inhibits agonist-induced transcription of PPARA-regulated genes, demonstrating target engagement in CLL cells [12]. Another inhibitor of PPARA, MK886, has also been shown to kill CLL cells [23]. However, the molecular mechanism of PPARA as a treatment target for CLL remains unclear.

Meanwhile, dozens of genes have also been suggested as CLL promoters or treatment targets in past years, and PPARA is one of them. According to its DC value, which indicates the significance of a vertex within a network, PPARA ranks 13th among 83 CLL promoters (as shown in Figure 4(a)). The result indicated that PPARA is more closely related to CLL and its TFS-related gene markers than most CLL promoter genes. Moreover, PPARA co-functions with most other CLL promoters in multiple pathways/gene groups related to the pathology of CLL. Our results support the importance of PPARA as a treatment target for CLL.

To explore the influence of PPARA in the prognosis and treatment of CLL at the molecular level, we first identified the CLL TFS-related gene markers by analyzing DNA–NGS data and clinical data of 86 CLL patients. Our results showed that mutations of four genes (EGR2, FBXW7, RPS15, and FOXO1) were directly related to shorter TFS, and six genes (NOTCH1, ATM, GNA12, KDM6A, KMT2A, and FBXW7) were linked to four clinical factors associated with shorter TFS, including CLL-IPI, del(11q), splenomegaly, and newly diagnosed platelets <100 × 10^9^/L. Moreover, mutations of two genes (EGR2 and GNA13) were associated with Richter's transformation of CLL. Altogether, our data suggested 10 CLL TFS-related gene markers. Interestingly, 6 out of these 10 CLL TFS-related gene markers have been implicated with CLL in previous studies (Figure 3), and 1 gene (NOTCH1) has also been reported as a CLL promoter in previous studies [23]. These results support the validity of the clinical/NGS data analysis.

Literature data mining also revealed 83 CLL promoters, which include PPARA. Out of these 83 genes, 69 genes present more or less linkage to the 10 CLL TFS-related gene markers, supporting their role in the prognosis and treatment of CLL. To note, ranked by DC, PPARA is superior to most of these genes by indexing No. 13. These results suggested the significance of PPARA as a treatment target for CLL.

Enrichment analysis showed that 91 out of 92 genes (CLL promoters and CLL TFS-related gene markers) were significantly enriched within 152 pathways/gene sets (False Discovery Rate (FDR) corrected *p*-value<3.4 × 10^−8^). Out of these pathways, PPARA was enriched within 12 pathways (Figure 5), and three of them were within the top 10 out of 152 pathways (FDR corrected *p*-value<1.03 × 10^−19^), which are linked to cell adhesion in general and leukocyte cell–cell adhesion. However, PPARA was not involved in the positive regulation of cell adhesion and cell migration, suggesting that PPARA might be involved in the negative cell contact and migration within the CLL pathology to influence the prognosis of CLL. It has been shown that CLL B cells induce alteration in cytoskeleton formation and vesicle transportation pathways in T cells by cell–cell contact [24], leading to T-cell dysfunction [25]. PPARA expression was found to be elevated in CLL patients and associated with an advanced disease stage [26]. There is evidence that activation of PPARA and PPARG has anti-inflammatory and immunomodulatory effects. Their agonists, such as troglitazone, inhibit the release of inflammatory cytokines from monocytes and induce apoptosis of T-lymphocytes [27].

The pathways where PPARA was enriched also include inflammation regulation, regulation of reactive oxygen species, regulation of cell differentiation, and their related pathways and gene groups (IL1B expression, peptide hormone, and oxygen response). All these pathways have been implicated in previous studies to play essential roles in the pathology of CLL [28–30]. Our findings indicate a potential association between PPARA and the prognosis of CLL, along with several possible underlying mechanisms.

It is worth pointing out that, together with PPARA, 70 out of the 92 CLL promoters and TFS-related genes (entities in the network presented in Figure 3) were enriched in these significant pathways mentioned above. Our results indicated that PPARA functionally collaborated with a large number of genetic markers to promote CLL. Other pathways not involving PPARA (140/153) may reflect the complex nature of CLL, and the relatively low EScore of PPARA suggests that the PPARA only plays roles in a piece of the whole pathology mechanism of CLL.

This study has several limitations. First, the genetic markers related to TFS length were identified through the analysis of NGS data from only 72 genes, which are used for routine screening of clinical CLL. Therefore, it is necessary to test more genes to explore CLL TFS length-related genetic markers thoroughly. Next, the regulation network of CLL TFS was constructed by integrating the findings of various independent studies. To confirm the accuracy of the network, it is recommended that all of the genetic markers be tested in the same batch of experiments.

## 5. Conclusion

Our findings reinforce the notion that PPARA is a crucial treatment target for CLL that is strongly linked to its TFS. PPARA collaborates with a considerable number of genetic markers and impacts the prognosis and TFS of CLL through several essential pathways, such as the regulation of cell adhesion, inflammation, reactive oxygen species, and cell differentiation.

## Figures and Tables

**Table 1:** Characteristics of 86 newly diagnosed chronic lymphoblastic leukemia patients.

Characteristics	*N* (%)
Sex	
Male	61 (70.93%)
Female	25 (29.07%)
Age (year)	
<45	9 (10.46%)
≥45, <65	51 (59.30%)
≥65	26 (30.23%)
CLL-IPI	
Low/intermediate risk	74 (86.05%)
High risk	12 (13.95%)
FISH	
del(13q)	12 (13.95%)
del(11q)	13 (15.11%)
del(17p)	5 (5.81%)
Trisomy 12	2 (2.32%)
TP53(NGS)	
TP53 mutation	5 (5.81%)
TP53 wt	78 (90.70%)
Unknown	3 (3.49%)
IGHV	
Mutated	61 (70.93%)
Unmutated	25 (29.07%)
Bulky disease	9 (10.465%)
Extranodal involvement	5 (5.814%)
Hepatomegaly	5 (5.814%)
Splenomegaly	42 (48.837%)
Initial WBC (×10^9^/L)	
>100	3 (3.488%)
50–100	15 (17.442%)
<50	68 (79.07%)
Initial HGB (g/L)	
>110	69 (80.233%)
90–110	11 (12.791%)
<90	6 (6.977%)
Initial PLT (×10^9^/L)	
≥100	77 (89.535%)
<100	9 (10.465%)
Initial LYM (%)	
>80	44 (51.163%)
50–80	31 (36.047%)
<50	11 (12.791%)
Initial LYMC (×10^9^/L)	
>100	3 (3.488%)
50–100	8 (9.302%)
<50	75 (87.209%)

## Data Availability

The data of this study are available from the corresponding author upon reasonable request.

## Supplementary Materials

Supplementary Materials Supplementary Figure 1 presents the spectrum of gene mutations of 86 CLL patients.

Supplementary Materials Supplementary Table 1 provides the results from univariate analysis and multivariate analysis of prognostic factor of chronic lymphocytic leukemia.

Supplementary Materials Supplementary Table 2 outlines the underlying information of each relationship presented in Figure 3, including the relation type, relation direction, source node, and target node.
