# Supplementary material for: Significance of PPARA as a Treatment Target for Chronic Lymphocytic Leukemia
Source: PPAR Res. 2023 Jun 26;2023:8456833. doi: 10.1155/2023/8456833 (PMC10317583; doi:10.1155/2023/8456833)
Supplement: Supplementary Materials — Supplementary Table 1 provides the results from univariate analysis and multivariate analysis of prognostic factor of chronic lymphocytic leukemia. [file 8456833.f2.pdf]

Supplementary Table 1 The results from univariate analysis and multivariate analysis of prognostic factor of chronic lymphocytic leukemia. The univariate analysis showed that 11 univariate variables with P<0.05 contributed to be poor prognostic factor. The factors with univariate analysis p<0.1 were included in the multivariate model for stepwise regression analysis. The variables in bold are statistically significant within the multivariable analysis.

**Factors**	**univariate analysis**			**Multivariable analysis**		
	P value	HR	95%CI	P value	HR	95%CI
Female	0.826	1.088	0.512-2.313			
Age						
<45		1				
≥45, <65	0.035	0.366	0.143-0.933			
≥65	0.082	0.389	0.135-1.126			
**CLL-IPI High risk**	**<0.001**	6.758	3.134-14.573	**0.025**	2.824	1.136-7.02
del(13q)	0.113	1.977	0.851-4.591			
**del(11q)**	**0.002**	3.388	1.546-7.424	**0.002**	3.772	1.615-8.807
del(17p)	<0.001	5.474	2.060-14.545			
TP53 mutation	0.059	2.765	0.963-7.938			
IGHV Unmutated	0.044	2.082	1.018-4.255			
Initial WBC（$*10^9$/L）						
≥100		1				
≥50, <100	0.938	0.941	0.206-4.307			
<50	0.114	0.308	0.071-1.329			
Initial HGB（g/L）						
≥110		1				
≥90, <110	0.3	1.679	0.63-4.474			
<90	<0.001	7.759	2.999-20.074			
**Initial PLT(<100*10^9/L)**	**<0.001**	6.373	2.764-14.691	**0.003**	**4.261**	**1.626-11.165**
Initial LYM (%)						
≥80		1				
≥50, <80	**0.022**	0.319	0.12-0.846			
<50	0.79	1.142	0.430-3.032			
Initial LYM($*10^9$/L)						
＞100		1				
≥50, <100	0.754	1.292	0.260-6.418			
＜50	0.143	0.339	0.080-1.444			
Bulky disease	0.035	2.625	1.071-6.432			
Extranodal	0.807	1.196	0.285-5.021			
Hepatomegaly	0.996	0.997	0.237-4.184			
**Splenomegaly**	**0.002**	3.651	1.629-8.181	**0.009**	**3.239**	**1.344-7.806**
EGR2 mutation	<0.001	5.5	2.2-14			
FBXW7 mutation	0.0014	4.4	1.8-11			
