# Supplementary material for: Significance of PPARA as a Treatment Target for Chronic Lymphocytic Leukemia
Source: PPAR Res. 2023 Jun 26;2023:8456833. doi: 10.1155/2023/8456833 (PMC10317583; doi:10.1155/2023/8456833)
Supplement: Supplementary Materials — Supplementary Table 2 outlines the underlying information of each relationship presented in Figure 3, including the relation type, relation direction, source node, and target node. [file 8456833.f3.pdf]

Supplementary Table 2 Details of each connection between 83 genes and CLL and the 10 CLL TFS-related genes, including the relation type, relation direction, source node, and target node.

negative Expression: ATM ---| TNF
positive Regulation: TNF --+> ATM
Regulation: ROR1 ---> FOXO1
ProtModification: ATM ---> FBXW7
positive Regulation: XIAP --+> chronic lymphocytic leukemia
positive MolTransport: NOTCH1 --+> IFNG
positive Regulation: CD40LG --+> chronic lymphocytic leukemia
positive Regulation: DLL4 --+> chronic lymphocytic leukemia
Expression: IL4 ---> EGR2
negative Regulation: GNA13 ---| MAPK1
Regulation: MIF ---> ATM
positive Expression: MAPK14 --+> EGR2
positive Regulation: EGR2 --+> IL4
Regulation: GNA12 ---> DAPK3
negative miRNAEffect: MIR223 ---| ATM
Regulation: MIR195 ---> FOXO1
positive Expression: EGR2 --+> PTHLH
positive DirectRegulation: MSI2 --+> KMT2A
negative Regulation: IRAK4 ---| KMT2A
negative Expression: NOTCH1 ---| PPARA
positive Expression: DLL4 --+> NOTCH1
positive DirectRegulation: DLL4 --+> NOTCH1
GeneticChange: chronic lymphocytic leukemia ---> EGR2
positive Expression: STAT3 --+> NOTCH1
positive Expression: FOXO1 --+> IL2
Expression: IL2 ---> FOXO1
positive Expression: NOTCH1 --+> DLL4
positive Regulation: EDNRA --+> GNA12
positive Regulation: PTHLH --+> chronic lymphocytic leukemia
Expression: NOTCH1 ---> FOXO1
DirectRegulation: NOTCH1 ---> FOXO1
positive MolTransport: KMT2A --+> GSK3B
positive Expression: TNFSF13B --+> NOTCH1
Regulation: FOXO1 ---> DLL4
negative Regulation: KMT2A ---| PPARA
Expression: PPARA ---> KMT2A
Regulation: NOTCH1 ---> CXCL13
negative miRNAEffect: MIR182 ---| FBXW7
Regulation: FBXW7 ---> PPARA
negative Regulation: FOXO1 ---| MAPK1
positive Regulation: CD44 --+> FOXO1
Regulation: ATM ---> MAPK1
Expression: PPARA ---> FOXO1
Expression: NOTCH1 ---> PPARA
Expression: NOTCH1 ---> XIAP
Regulation: NOTCH1 ---> GSK3B
Expression: FBXW7 ---> TNF
negative Regulation: NOTCH1 ---| ATM
negative Expression: VEGFA ---| ATM

Regulation: KMT2A ---> IL4
Regulation: ATM ---> RPS15
positive Regulation: IL2 --+> chronic lymphocytic leukemia
Expression: ATM ---> CYCS
positive Regulation: NAMPT --+> chronic lymphocytic leukemia
GeneticChange: chronic lymphocytic leukemia ---> NOTCH1
positive Expression: NOTCH1 --+> TNFSF13B
Biomarker: chronic lymphocytic leukemia ---> NOTCH1
Regulation: THPO ---> ATM
positive Regulation: IL4 --+> FOXO1
positive Expression: EGR2 --+> TNFSF10
CellExpression: chronic lymphocytic leukemia ---- NOTCH1
Regulation: EGFR ---> FOXO1
positive Regulation: NOTCH1 --+> chronic lymphocytic leukemia
positive Regulation: TNFSF14 --+> chronic lymphocytic leukemia
positive Regulation: MIR363 --+> chronic lymphocytic leukemia
positive Regulation: OGFOD1 --+> chronic lymphocytic leukemia
ProtModification: ATM ---> FOXO1
PromoterBinding: KMT2A ---> TNF
positive Regulation: MIR182 --+> chronic lymphocytic leukemia
Regulation: ICAM1 ---> FOXO1
positive Expression: NOTCH1 --+> VEGFA
positive Expression: VEGFA --+> NOTCH1
positive PromoterBinding: FOXO1 --+> ICAM1
DirectRegulation: MSI2 ---> KMT2A
positive Regulation: CSNK1D --+> chronic lymphocytic leukemia
positive Regulation: ZAP70 --+> chronic lymphocytic leukemia
positive Expression: KMT2A --+> CD44
negative Regulation: HIF1A ---| EGR2
positive Regulation: CD27 --+> chronic lymphocytic leukemia
positive Regulation: TNFSF10 --+> chronic lymphocytic leukemia
positive Regulation: IL4 --+> chronic lymphocytic leukemia
positive Expression: FOXO1 --+> CD40
positive Expression: NOTCH1 --+> IFNG
MolTransport: NOTCH1 ---> IFNG
Regulation: CD40 ---> FOXO1
Regulation: IL24 ---> FOXO1
QuantitativeChange: chronic lymphocytic leukemia ---> KMT2A
positive Expression: PIK3CG --+> NOTCH1
Expression: NOTCH1 ---> PIK3CG
CellExpression: KMT2A ---- chronic lymphocytic leukemia
positive Regulation: FOXO1 --+> WNT5A
GeneticChange: chronic lymphocytic leukemia ---> KMT2A
Regulation: GNA13 ---> MAPK1
Expression: FOXO1 ---> BCL2L1
negative Expression: NOTCH1 ---| BCL2L12
negative PromoterBinding: FOXO1 ---| IFNG
negative miRNAEffect: MIR223 ---| FBXW7
negative MolTransport: KMT2A ---| MIF
negative Expression: CD27 ---| FOXO1
positive Regulation: MIF --+> KMT2A
positive Expression: KMT2A --+> BCL2L1
negative Expression: MAPK1 ---| FOXO1
ProtModification: MAPK1 ---> FOXO1

negative Expression: FOXO1 ---| HIF1A
positive Expression: CD40 --+> EGR2
Expression: CXCR5 ---> FOXO1
negative Expression: KMT2A ---| MIF
positive PromoterBinding: HIF1A --+> FOXO1
Expression: EGR2 ---> IFNG
positive Expression: IFNG --+> EGR2
negative MolTransport: EGR2 ---| IFNG
Regulation: ATM ---> STAT3
Expression: FBXW7 ---> VEGFA
negative Expression: FBXW7 ---| HIF1A
Regulation: KMT2A ---> chronic lymphocytic leukemia
positive Regulation: HAVCR2 --+> chronic lymphocytic leukemia
Regulation: FOXO1 ---> MAPK1
positive Regulation: ZFYVE19 --+> chronic lymphocytic leukemia
positive Regulation: NEDD9 --+> chronic lymphocytic leukemia
positive Expression: CXCL12 --+> FOXO1
Regulation: IFNG ---> FOXO1
positive Expression: NOTCH1 --+> ICAM1
positive Regulation: ICAM1 --+> NOTCH1
positive Regulation: VEGFA --+> chronic lymphocytic leukemia
positive Regulation: EDNRA --+> chronic lymphocytic leukemia
positive Regulation: SYK --+> chronic lymphocytic leukemia
positive Expression: NOTCH1 --+> PDCD1
positive Regulation: HDAC6 --+> chronic lymphocytic leukemia
negative Expression: NOTCH1 ---| TCF3
positive Regulation: CXCL12 --+> chronic lymphocytic leukemia
positive Regulation: ZNF215 --+> chronic lymphocytic leukemia
positive PromoterBinding: TCF3 --+> NOTCH1
Regulation: PTHLH ---> FOXO1
Regulation: NOTCH1 ---> MAPK14
Regulation: KMT2A ---> MAPK1
PromoterBinding: RELA ---> EGR2
Regulation: EDN1 ---> NOTCH1
positive PromoterBinding: FOXO1 --+> CXCR4
positive Regulation: PIK3CG --+> chronic lymphocytic leukemia
Regulation: CXCR4 ---> FOXO1
negative Expression: GNA13 ---| DLL4
Regulation: EDN1 ---> GNA13
positive Regulation: PTHLH --+> NOTCH1
positive Expression: GNA13 --+> EDN1
negative miRNAEffect: MIR195 ---| FBXW7
positive Regulation: MIF --+> chronic lymphocytic leukemia
Regulation: MIR130A ---> ATM
Regulation: PTHLH ---> NOTCH1
positive Regulation: EGFR --+> GNA13
positive Expression: GNA12 --+> IL2
Binding: GNA13 ---- CXCR5
positive Regulation: NAMPT --+> ATM
Expression: IL4 ---> FOXO1
positive Expression: CXCL12 --+> NOTCH1
Regulation: IL21 ---> FOXO1
Regulation: NOTCH1 ---> CXCL12
Expression: TNFSF11 ---> FOXO1

PromoterBinding: FOXO1 ---> TNFSF11
Expression: IL4 ---> ATM
negative Expression: ATM ---| IL4
positive Expression: ATM --+> GSK3B
positive Expression: TNF --+> FOXO1
positive PromoterBinding: FOXO1 --+> TNF
positive Regulation: EGR2 --+> CXCR5
positive Regulation: ATM --+> XIAP
Regulation: TNFSF10 ---> ATM
negative Regulation: ATM ---| TNFSF10
positive Expression: NOTCH1 --+> WNT5A
Binding: NOTCH1 ---- DLL4
Regulation: IFNG ---> NOTCH1
positive Expression: EGR2 --+> TNF
positive Expression: TNF --+> EGR2
Expression: FOXO1 ---> CXCR4
negative Expression: FBXW7 ---| BCL2L1
negative miRNAEffect: MIR15A ---| FOXO1
negative Regulation: NOTCH1 ---| KCNA3
positive Regulation: KCNA3 --+> NOTCH1
positive Regulation: MIR223 --+> NOTCH1
positive PromoterBinding: KMT2A --+> TNF
positive MolTransport: KMT2A --+> TNF
Expression: NOTCH1 ---> MIR223
positive Regulation: TNF --+> KMT2A
positive DirectRegulation: CXCR4 --+> GNA13
PromoterBinding: FOXO1 ---> EDN1
ProtModification: ATM ---> CSNK1D
Expression: ATM ---> IFNG
positive Regulation: IFNG --+> ATM
positive Regulation: KDM6A --+> FBXW7
Expression: FOXO1 ---> NAMPT
Regulation: EDN1 ---> FOXO1
positive Expression: MAPK14 --+> NOTCH1
negative Expression: KMT2A ---| CXCL12
positive Regulation: NOTCH1 --+> MAPK14
Expression: NOTCH1 ---> STAT6
Regulation: EGR2 ---> MAPK1
positive Expression: IL4 --+> NOTCH1
positive Expression: NOTCH1 --+> BCL2L1
negative Regulation: NOTCH1 ---| EDNRA
positive MolTransport: IL24 --+> ATM
Expression: GSK3B ---> FOXO1
ProtModification: FBXW7 ---> PDCD1
positive Regulation: MSI2 --+> NOTCH1
Regulation: TNFSF13B ---> FOXO1
positive Expression: MAPK1 --+> EGR2
positive Expression: FOXO1 --+> TNFSF13B
Expression: KDM6A ---> CD44
Expression: EGR2 ---> VEGFA
Expression: KDM6A ---> ICAM1
positive Expression: IL4 --+> EGR2
negative Regulation: IL7 ---| FOXO1
positive Expression: KMT2A --+> HIF1A

DirectRegulation: EGFR ---> ATM
positive Regulation: CX3CL1 --+> chronic lymphocytic leukemia
negative Expression: ATM ---| IL2
positive Regulation: TNFSF13B --+> chronic lymphocytic leukemia
Expression: NOTCH1 ---> TNFSF10
positive Regulation: CXCR4 --+> chronic lymphocytic leukemia
Regulation: TNFSF10 ---> NOTCH1
positive Expression: MAPK1 --+> NOTCH1
Expression: NAMPT ---> NOTCH1
Expression: MIF ---> FBXW7
positive Regulation: NOTCH1 --+> IL7
positive Regulation: EDN1 --+> ATM
positive Regulation: PDCD1 --+> chronic lymphocytic leukemia
Expression: GNA12 ---> IFNG
negative miRNAEffect: MIR363 ---| FBXW7
DirectRegulation: WNT5A ---> NOTCH1
positive Regulation: CXCR4 --+> GNA12
negative Expression: STAT6 ---| NOTCH1
Regulation: KMT2A ---> NOTCH1
ProtModification: GSK3B ---> FBXW7
negative Regulation: VEGFA ---| ATM
negative Expression: GSK3B ---| FBXW7
negative Expression: ATM ---| VEGFA
positive Regulation: PTX3 --+> chronic lymphocytic leukemia
Expression: NOTCH1 ---> ITGA4
positive Regulation: r_Mir16 --+> chronic lymphocytic leukemia
Regulation: KMT2A ---> RELA
Regulation: GNA12 ---> MAPK14
Regulation: ATM ---> EGFR
Regulation: NOTCH1 ---> MAPK1
ProtModification: MAPK1 ---> FBXW7
positive Expression: FOXO1 --+> NAMPT
Expression: MAPK1 ---> FBXW7
PromoterBinding: FOXO1 ---> NAMPT
positive Regulation: ROR1 --+> chronic lymphocytic leukemia
positive Expression: NOTCH1 --+> XIAP
DirectRegulation: NOTCH1 ---> XIAP
Regulation: NAMPT ---> FOXO1
Regulation: FBXW7 ---> MAPK1
positive Expression: IL7 --+> NOTCH1
negative Expression: GNA12 ---| TNFSF11
Expression: ATM ---> CD44
Expression: KDM6A ---> TNF
Regulation: FBXW7 ---> EGFR
Regulation: TNF ---> KDM6A
Regulation: GNA13 ---> RELA
positive Regulation: WNT16 --+> chronic lymphocytic leukemia
PromoterBinding: FOXO1 ---> VEGFA
negative ProtModification: GSK3B ---| FOXO1
Regulation: VEGFA ---> FOXO1
Expression: FOXO1 ---> STAT3
ProtModification: FBXW7 ---> NOTCH1
Binding: NOTCH1 ---- XIAP
Regulation: IL2 ---> EGR2

negative miRNAEffect: MIR182 ---| FOXO1
Expression: EGR2 ---> IL2
positive Regulation: GSTP1 --+> chronic lymphocytic leukemia
positive Regulation: TNFSF11 --+> GNA13
Regulation: ATM ---> MIR182
positive Regulation: MIR130A --+> chronic lymphocytic leukemia
negative miRNAEffect: MIR182 ---| ATM
Regulation: GSK3B ---> KMT2A
Expression: IL2 ---> NOTCH1
positive Expression: NOTCH1 --+> RELA
Regulation: KMT2A ---> GSK3B
positive MolTransport: NOTCH1 --+> RELA
ProtModification: RELA ---> NOTCH1
Regulation: TNFSF11 ---> NOTCH1
positive Regulation: IRAK4 --+> chronic lymphocytic leukemia
PromoterBinding: RELA ---> NOTCH1
Expression: FOXO1 ---> IL2
Regulation: FOXO1 ---> RELA
Regulation: EGR2 ---> GSK3B
Expression: TNFSF11 ---> EGR2
Regulation: ATM ---> FOXO1
positive Expression: NOTCH1 --+> IL4
positive PromoterBinding: STAT3 --+> NOTCH1
positive Regulation: WNT3 --+> chronic lymphocytic leukemia
positive Regulation: IL4 --+> NOTCH1
positive Regulation: HIF1A --+> chronic lymphocytic leukemia
positive Regulation: CXCL13 --+> chronic lymphocytic leukemia
Regulation: GNA12 ---> EGFR
positive Expression: GNA12 --+> VEGFA
positive Expression: NAMPT --+> NOTCH1
Expression: CD40LG ---> ATM
negative Expression: GSK3B ---| EGR2
positive Regulation: IL21 --+> chronic lymphocytic leukemia
negative Expression: FOXO1 ---| TNFSF11
Expression: MAPK1 ---> EGR2
Expression: PTPN22 ---> ATM
positive DirectRegulation: TCF3 --+> FOXO1
positive PromoterBinding: TCF3 --+> FOXO1
Regulation: WNT3 ---> NOTCH1
positive Regulation: EGFR --+> chronic lymphocytic leukemia
Regulation: GNA13 ---> GSK3B
Regulation: EGR2 ---> CD44
positive Regulation: STAT6 --+> chronic lymphocytic leukemia
negative Regulation: PIK3CD ---| FOXO1
positive Regulation: BCL2L1 --+> chronic lymphocytic leukemia
positive Regulation: GNA12 --+> GSK3B
positive Regulation: NOTCH1 --+> CXCL12
positive Regulation: CYCS --+> chronic lymphocytic leukemia
positive Regulation: THPO --+> EGR2
positive Regulation: PIK3CD --+> chronic lymphocytic leukemia
positive Regulation: RELA --+> chronic lymphocytic leukemia
Expression: NOTCH1 ---> TNF
positive Regulation: GNA12 --+> STAT3
positive Regulation: KCNA3 --+> chronic lymphocytic leukemia

positive Regulation: CD70 --+> chronic lymphocytic leukemia
Expression: CD40LG ---> KMT2A
Expression: IL4 ---> NOTCH1
negative Expression: NOTCH1 ---| ZAP70
positive Expression: GNA13 --+> VEGFA
positive PromoterBinding: STAT3 --+> FOXO1
negative Regulation: FBXW7 ---| DLL4
positive Expression: FOXO1 --+> CYCS
Regulation: IL4 ---> KDM6A
Binding: HDAC6 ---- FOXO1
positive Regulation: EGFR --+> FOXO1
positive Regulation: NOTCH1 --+> CXCL13
PromoterBinding: HIF1A ---> FOXO1
ProtModification: ATM ---> TCF3
Regulation: CD40 ---> ATM
positive Expression: KDM6A --+> HAVCR2
positive Expression: NOTCH1 --+> IL2
positive Regulation: WNT16 --+> GNA12
negative Expression: EGFR ---| NOTCH1
positive Regulation: CD44 --+> chronic lymphocytic leukemia
positive Expression: NOTCH1 --+> EGFR
Expression: NOTCH1 ---> WNT3
positive Regulation: GSTM1 --+> chronic lymphocytic leukemia
QuantitativeChange: chronic lymphocytic leukemia ---> FBXW7
GeneticChange: chronic lymphocytic leukemia ---> FBXW7
positive Regulation: FBXW7 --+> CD40LG
negative miRNAEffect: MIR363 ---| NOTCH1
negative miRNAEffect: MIR182 ---| GNA13
PromoterBinding: FOXO1 ---> PPARA
positive Expression: KDM6A --+> TNFSF10
positive Regulation: STAT6 --+> EGR2
positive Regulation: NME2 --+> chronic lymphocytic leukemia
Regulation: FBXW7 ---> STAT3
positive Expression: KDM6A --+> PDCD1
positive Expression: NOTCH1 --+> CD44
MolTransport: PIK3CG ---> FOXO1
positive Expression: KDM6A --+> WNT3
positive Regulation: MAPK1 --+> chronic lymphocytic leukemia
positive Regulation: ATM --+> BCL2L1
Expression: NOTCH1 ---> IFNG
positive Expression: KDM6A --+> IFNG
positive Expression: NOTCH1 --+> MAPK1
Expression: NOTCH1 ---> CXCL12
positive PromoterBinding: FOXO1 --+> PDCD1
Expression: NOTCH1 ---> CXCR4
positive Expression: NOTCH1 --+> HIF1A
positive PromoterBinding: HIF1A --+> NOTCH1
positive Expression: FOXO1 --+> IL4
PromoterBinding: KMT2A ---> WNT5A
negative Regulation: NOTCH1 ---| GSK3B
positive Expression: GNA12 --+> HIF1A
Expression: GSK3B ---> NOTCH1
ProtModification: GSK3B ---> NOTCH1
negative Regulation: FOXO1 ---| STAT6

positive Regulation: CXCL12 --+> GNA13
positive Regulation: PPARA --+> chronic lymphocytic leukemia
Regulation: VEGFA ---> EGR2
negative DirectRegulation: NOTCH1 ---| ATM
positive Regulation: GNA13 --+> EGFR
ProtModification: ATM ---> HIF1A
positive Regulation: MAPK1 --+> ATM
positive Regulation: ATM --+> MAPK1
positive Regulation: WNT5A --+> chronic lymphocytic leukemia
positive Regulation: ITGA4 --+> chronic lymphocytic leukemia
Expression: FBXW7 ---> EGFR
PromoterBinding: STAT3 ---> NOTCH1
positive Regulation: NOTCH1 --+> STAT3
positive Regulation: EDN1 --+> chronic lymphocytic leukemia
positive Regulation: GNA13 --+> MAPK14
positive Regulation: GNA12 --+> MAPK14
Expression: CD44 ---> FOXO1
ProtModification: KDM6A ---> NOTCH1
PromoterBinding: STAT3 ---> FOXO1
ProtModification: MAPK14 ---> FOXO1
negative Expression: CD40 ---| KMT2A
positive Expression: MAPK14 --+> FOXO1
positive Regulation: SEMA4D --+> chronic lymphocytic leukemia
ProtModification: FOXO1 ---> GSK3B
Expression: TNF ---> NOTCH1
Expression: ATM ---> RELA
DirectRegulation: TNF ---> NOTCH1
ProtModification: GSK3B ---> FOXO1
Expression: FOXO1 ---> SYK
positive Expression: TCF3 --+> FOXO1
negative Expression: FBXW7 ---| RELA
PromoterBinding: RELA ---> FBXW7
positive Expression: NOTCH1 --+> STAT3
positive Regulation: STAT3 --+> EGR2
Regulation: EGR2 ---> STAT3
Regulation: EGR2 ---> NOTCH1
negative Expression: NOTCH1 ---| EGR2
negative Expression: ATM ---| IFNG
positive Regulation: CD40 --+> chronic lymphocytic leukemia
Regulation: ATM ---> RELA
positive Expression: NOTCH1 --+> TNF
positive MolTransport: KDM6A --+> RELA
positive Regulation: TNF --+> chronic lymphocytic leukemia
Expression: NOTCH1 ---> MAPK1
GeneticChange: chronic lymphocytic leukemia ---> RPS15
ProtModification: HDAC6 ---> FOXO1
positive Regulation: TNFSF11 --+> chronic lymphocytic leukemia
positive Regulation: MIR223 --+> chronic lymphocytic leukemia
Regulation: ATM ---> GSK3B
Regulation: CSNK1D ---> FOXO1
Regulation: HDAC6 ---> EGR2
positive Regulation: KMT2A --+> MAPK1
negative miRNAEffect: MIR223 ---| FOXO1
Regulation: SYK ---> FOXO1

positive Regulation: DAPK3 --+> chronic lymphocytic leukemia
negative miRNAEffect: MIR223 ---| KMT2A
positive Expression: EGFR --+> EGR2
Expression: KMT2A ---> VEGFA
Expression: EGFR ---> KDM6A
positive MolTransport: NOTCH1 --+> CXCL12
Regulation: GNA12 ---> MAPK1
positive Expression: NOTCH1 --+> PPARA
positive Regulation: MIR663B --+> chronic lymphocytic leukemia
Regulation: SDF-1alpha ---> FOXO1
Expression: FOXO1 ---> SDF-1alpha
negative Regulation: WNT5A ---| EGR2
positive Regulation: ATM --+> CXCR4
negative Expression: EGR2 ---| IL21
Regulation: KMT2A ---> MAPK14
Regulation: CD40LG ---> FOXO1
Expression: MAPK14 ---> EGR2
Regulation: ATM ---> KMT2A
positive Regulation: MSI2 --+> chronic lymphocytic leukemia
positive Regulation: ATM --+> MAPK14
positive Regulation: CD40LG --+> ATM
negative Expression: ATM ---| CD27
ProtModification: MAPK1 ---> RPS15
positive Regulation: GNA13 --+> STAT3
negative Regulation: KMT2A ---| MAPK14
positive Regulation: MAPK14 --+> KMT2A
positive Expression: KDM6A --+> CXCR4
positive PromoterBinding: STAT3 --+> KDM6A
negative Expression: GNA12 ---| DLL4
negative Expression: GNA12 ---| IL4
positive Expression: KMT2A --+> CXCR4
positive Regulation: BCL2L12 --+> chronic lymphocytic leukemia
positive Regulation: IL7 --+> chronic lymphocytic leukemia
positive Regulation: CD47 --+> chronic lymphocytic leukemia
negative Regulation: IFNG ---| NOTCH1
negative Expression: FOXO1 ---| IFNG
positive Regulation: STAT3 --+> chronic lymphocytic leukemia
negative Regulation: PPARA ---| FOXO1
positive Regulation: EGR2 --+> TNFSF11
positive Regulation: BCL2A1 --+> chronic lymphocytic leukemia
positive MolTransport: NOTCH1 --+> VEGFA
negative Expression: FOXO1 ---| VEGFA
positive Expression: IL2 --+> FOXO1
positive Regulation: IFNG --+> chronic lymphocytic leukemia
positive Expression: EGR2 --+> PDCD1
Expression: IL24 ---> NOTCH1
negative miRNAEffect: MIR182 ---| EGR2
positive Regulation: SDF-1alpha --+> chronic lymphocytic leukemia
positive Expression: FOXO1 --+> RELA
Expression: KMT2A ---> TNFSF11
positive Regulation: NOTCH1 --+> THPO
ProtModification: MAPK14 ---> FBXW7
Binding: NOTCH1 ---- FOXO1
negative Expression: MAPK14 ---| FBXW7

positive Regulation: TCF3 --+> chronic lymphocytic leukemia
negative Regulation: ATM ---| HDAC6
PromoterBinding: KMT2A ---> IFNG
positive Regulation: CXCR5 --+> chronic lymphocytic leukemia
positive Regulation: FOXO1 --+> NOTCH1
Expression: HDAC6 ---> NOTCH1
positive Regulation: IL24 --+> chronic lymphocytic leukemia
positive Regulation: MAPK14 --+> chronic lymphocytic leukemia
positive Regulation: MIR195 --+> chronic lymphocytic leukemia
positive Regulation: GSK3B --+> chronic lymphocytic leukemia
negative Expression: FBXW7 ---| NOTCH1
positive Regulation: MIR15A --+> chronic lymphocytic leukemia
Expression: MAPK1 ---> KMT2A
negative ProtModification: FBXW7 ---| NOTCH1
positive Regulation: ICAM1 --+> chronic lymphocytic leukemia
Expression: FOXO1 ---> VEGFA
Regulation: PPARA ---> chronic lymphocytic leukemia
positive Expression: FOXO1 --+> TNF
Expression: NOTCH1 ---> TNFSF11
positive Regulation: WNT5A --+> FOXO1
Expression: FOXO1 ---> WNT5A
Expression: NOTCH1 ---> STAT3
positive Expression: TNFSF11 --+> NOTCH1
Binding: KDM6A ---- KMT2A
positive Regulation: WNT16 --+> GNA13
positive Regulation: PTPN22 --+> chronic lymphocytic leukemia
positive Regulation: ATM --+> BCL2A1
positive Regulation: GNA13 --+> WNT16
ProtModification: CYCS ---> NOTCH1
positive Expression: NOTCH1 --+> CYCS
positive Regulation: EDN1 --+> GNA12
negative QuantitativeChange: chronic lymphocytic leukemia ---| ATM
Regulation: ATM ---> chronic lymphocytic leukemia
GeneticChange: chronic lymphocytic leukemia ---> ATM
Biomarker: chronic lymphocytic leukemia ---> ATM
positive Regulation: THPO --+> chronic lymphocytic leukemia
positive Expression: NOTCH1 --+> CXCR4
positive Regulation: GSTP1 --+> ATM
positive DirectRegulation: NOTCH1 --+> XIAP
positive PromoterBinding: FOXO1 --+> TNFSF10
