# Supplementary material for: Significance of PPARA as a Treatment Target for Chronic Lymphocytic Leukemia
Source: PPAR Res. 2023 Jun 26;2023:8456833. doi: 10.1155/2023/8456833 (PMC10317583; doi:10.1155/2023/8456833)
Supplement: Supplementary Materials — Supplementary Figure 1 presents the spectrum of gene mutations of 86 CLL patients. [file 8456833.f1.pdf]

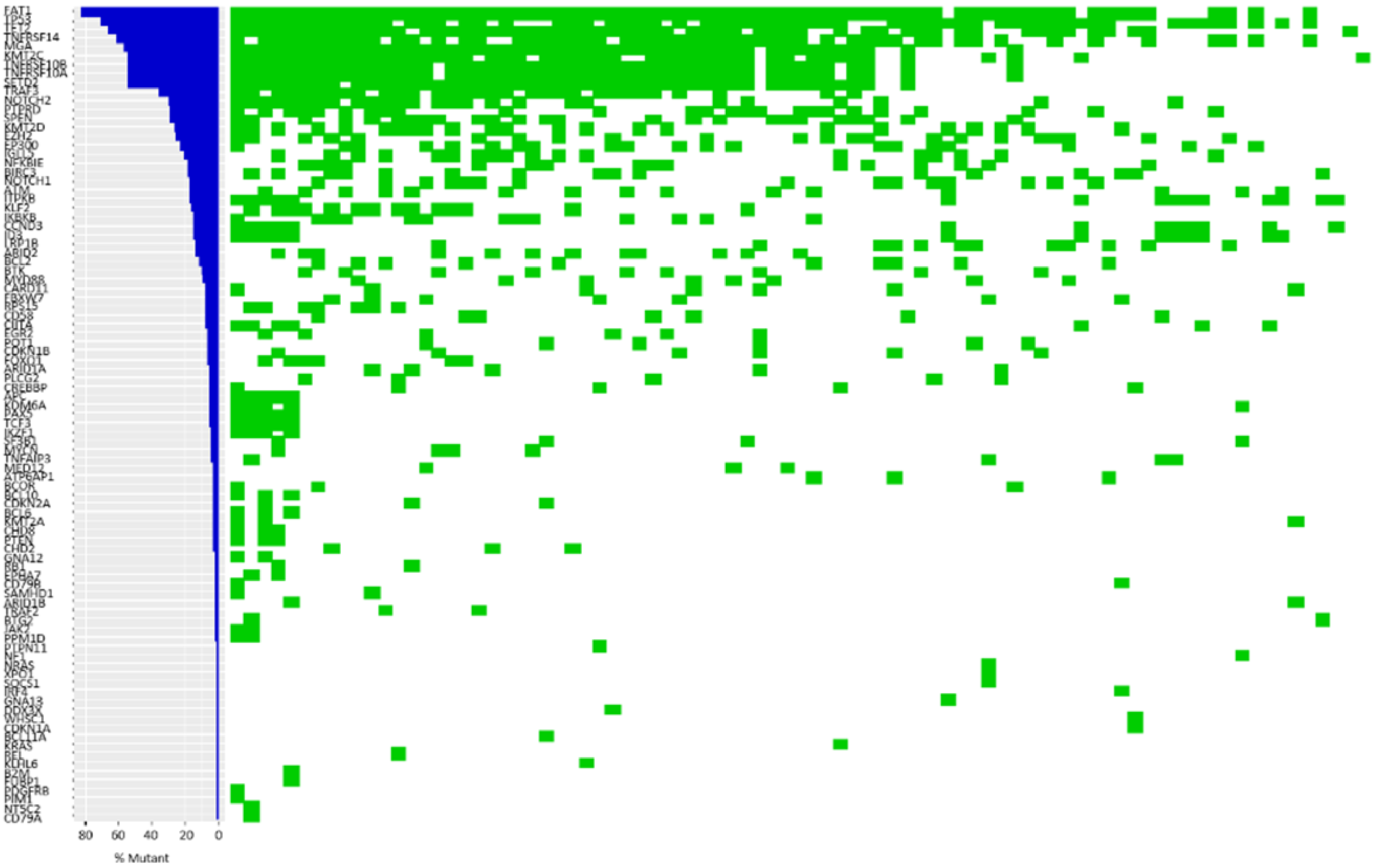

Supplementary Figure 1 The spectrum of gene mutations of 86 CLL patients
